# Factors associated with glomerular filtration rate variation in primary hyperparathyroidism after parathyroidectomy

**DOI:** 10.3906/sag-1806-181

**Published:** 2019-02-11

**Authors:** Mustafa ÇALIŞKAN, Muhammed KIZILGÜL, Selvihan BEYSEL, Bekir UÇAN, Fatih AKCAN, Mümtaz TAKIR, Mustafa ÖZBEK, Erman ÇAKAL

**Affiliations:** 1 Department of Endocrinology and Metabolism, University of Health Sciences,Dışkapı Training and Research Hospital, Ankara Turkey; 2 Department of Otorhinolaryngology, School of Medicine, Düzce University, Düzce Turkey; 3 Department of Endocrinology and Metabolism, Medeniyet University, Göztepe Training and Research Hospital, İstanbul Turkey

**Keywords:** Primary hyperparathyroidism, predictors, renal failure, parathyroidectomy

## Abstract

**Background/aim:**

Prolonged hypercalcemia impairs renal function, and a reduced glomerular filtration rate (GFR) is typical in advanced primary hyperparathyroidism (PHPT). There are scarce data related to predictors of renal impairment in patients with PHPT. Hence, we aimed to evaluate changes in kidney function in PHPT patients after parathyroidectomy (PTX) and identify factors associated with GFR variation in these patients.

**Materials and methods:**

One hundred and twenty-five patients with PHPT who underwent surgery between 2012 and 2014 were enrolled in the study. Patients were divided into two groups according to GFR values: patients whose GFR was lower than 60 mL/min/1.73 m2 and higher than 60 mL/min/1.73 m2. Demographic and laboratory parameters were compared before and 6 months after parathyroidectomy.

**Results:**

Prevalence of antihypertensive drug users and patients with renal cysts and parathormone (PTH) and alkaline phosphatase levels were higher in patients with GFR of ≥60 than in GFR of <60 mL/min/1.73 m2 (P < 0.05). Systolic BP, uric acid, and magnesium were decreased in patients with GFR of ≥60, but GFR did not change in the two groups after parathyroidectomy. After parathyroidectomy, calcium and PTH decreased but 25(OH)D3 and phosphorus increased in the two groups. In multiple regression analysis, age, calcium, and baseline GFR were independent predictors of GFR variation. Parathyroid adenoma volume and urinary calcium were not independent predictors of GFR change.

**Conclusion:**

Older age, higher preoperative calcium, and GFR were factors associated with GFR increase in PHPT patients after parathyroidectomy. Further renal impairment was prevented by parathyroidectomy in PHPT patients.

## 1. Introduction

Primary hyperparathyroidism (PHPT), characterized by overactivation of parathyroid glands causing excessive release of parathyroid hormone, is a relatively common endocrine disorder, with a prevalence of 1–7 cases per 1000 adults (1,2). Prolonged hypercalcemia impairs renal function, and a reduced glomerular filtration rate (GFR) is typical in advanced PHPT (3,4). Hypercalcemia-induced diuresis, nephrocalcinosis, and nephrolithiasis are considered to be possible mechanisms causing chronic kidney disease (CKD) in PHPT; however, the exact pathogenesis is still unclear (4). Most studies reported rates of the prevalence of CKD as approximately 16%–17% when an estimated glomerular filtration rate (eGFR) cutoff below 60 mL/min per 1.73 m2 is taken into consideration (5–7). Renal impairment is considered as a surgical indication for PHPT (8,9) since surgery might preserve renal function in these patients (9). However, some studies showed no improvement in renal function after parathyroidectomy (PTX) (3,4,10–12). Furthermore, recent randomized controlled trials conducted primarily with mild asymptomatic PHPT patients could not demonstrate an improvement in renal function after PTX (13–16). There are scarce data related to predictors of renal impairment in patients with PHPT. Hence, we aimed to evaluate changes in kidney function in PHPT patients after PTX and identify factors associated with GFR variation in these patients. 

## 2. Materials and methods

### 2.1. Patient selection

One hundred and twenty-five patients with PHPT who underwent surgery in Dışkapı Teaching and Research Hospital between 2012 and 2014 was enrolled in the study. The study was approved by the Ethical Committee of Düzce University (Date: 23.10.2017, No: 2017/139) and written informed consent of participants was obtained before the study. Patients with a complete medical history, serum biochemistry and urinalysis results, ultrasonography of the parathyroids and kidneys, and follow-up examination after curative parathyroidectomy were included in the study. Patients with multiple endocrine neoplasia, parathyroid cancer, thyroid cancer, hyperparathyroidism-jaw tumor syndrome, or interference with calcium and vitamin D metabolism for at least 2 weeks before hospital admission were excluded. PHPT was diagnosed by demonstrating persistent hypercalcemia in the presence of inappropriately normal or elevated PTH concentrations (8). Asymptomatic primary hyperparathyroidism described patients who lacked obvious signs and symptoms associated with either elevated calcium or parathyroid hormone. The criteria for surgery were in agreement with guidelines applied at the time of the diagnosis (8). Six patients had double adenoma and 119 patients had single adenoma.

Patients were divided into two groups according to GFR values: patients whose GFR was lower than 60 mL/min/1.73 m2 and higher than 60 mL/min/1.73 m2. Demographic and laboratory parameters were compared before and 6 months after parathyroidectomy in both groups. GFR variation is defined as the difference between GFR levels at basal and 6 months after parathyroidectomy.

### 2.2. Clinical, biochemical, and hormonal measurements

Weight, height, circumferences of waist (WC) and hip (HC), and systolic and diastolic blood pressure (BP) were measured. All patients underwent a biochemical and hormonal examination including serum glucose, albumin, total calcium, phosphorus, creatinine, total cholesterol, triglyceride, low-density lipoprotein cholesterol (LDL-C), and high-density lipoprotein cholesterol (HDL-C) levels the morning after an overnight fast, using colorimetric methods. The concentration of plasma parathyroid hormone (PTH) was measured using an intact PTH assay (chemiluminescent immunoassay with an Immulite 2000; normal range 12–65 pg/mL). Plasma 25-OH vitamin D was measured via radioimmune assay. Vitamin D deficiency was assessed by classifying serum 25(OH)D3 concentrations as follows: ≤ 10 ng/mL , severe; 11–20 ng/mL, moderate; and 21–30 ng/mL, mild (17).

The CKD-EPI creatinine equation was used for measurement of glomerular filtration rate as follows (18): 141 × min(Scr/κ,1)α × max(Scr/κ,1) × 1.209 × 0.993Age × 1.018 [if female] × 1.159 [if black], where SCr is serum creatinine (in mg/dL), k is 0.7 for women and 0.9 for males, α is –0.329 for women and –0.411 for males, min is the minimum of SCr/k or 1, and max is the maximum of SCr/k or 1.

Parathyroid ultrasonography (US) was performed using high-resolution B-mode ultrasound images (EUB 7000 HV; Hitachi, Tokyo, Japan) with a 13-MHz linear array transducer. The volume of parathyroid adenoma was calculated by the ellipsoid model formula (length × thickness × width × 0.52) (19).

### 2.3. Statistical analysis

Statistical analysis was performed using SPSS 18.0 (SPSS Inc., Chicago, IL, USA). Descriptive analyses were expressed as mean ± standard deviation (SD), percentages (%), and beta (β). Kolmogorov–Smirnov or Shapiro–Wilk W tests were used for normality. Chi-square tests or Fisher exact tests, where appropriate, were used for categorical variables. The Student t-test for normally distributed continuous variables and Mann–Whitney U test for continuous variables that were not normally distributed were used. The paired samples t-test for normally distributed continuous variables and Wilcoxon test for continuous variables that were not normally distributed were analyzed before and after parathyroidectomy. Multiple linear regression was used to examine factors associated with GFR variations. P < 0.05 was considered statistically significant. 

## 3. Results

The mean age of PHPT patients was 53.93 ± 10.67 years. Parathyroid adenoma volume was 1.17 ± 2.16 cm3 and urinary calcium excretion was 394.49 ± 184.40 mmol/dL. After parathyroidectomy, blood pressures, serum calcium, uric acid, magnesium, PTH, and ALP decreased, but 25(OH)D3 and phosphorus increased in PHPT patients (P < 0.05) (Table 1). Patients were divided into two groups according to GFR values: patients with values lower than 60 mL/min/1.73 m2 and patients with values higher than 60 mL/min/1.73 m2. Mean age was higher in patients whose GFR was <60 mL/min/1.73 m2 (66.12 ± 7.05 vs. 52.13 ± 9.93, P < 0.0001). Prevalences of patients with diabetes, renal stones, and thiazide usage were similar between groups (P > 0.05). BMI and blood pressures were similar in the two groups. Prevalence of patients with hypertension and renal cysts were higher in patients with GFR of <60 mL/min/1.73 m2 (P < 0.05). PTH and ALP levels were higher in patients with GFR of ≥60 than <60 mL/min/1.73 m2 (P < 0.05) (Table 2). Systolic BP, uric acid, and magnesium decreased in patients with GFR ≥60, but GFR did not change in the two groups after parathyroidectomy. After parathyroidectomy, serum calcium and PTH decreased, but 25(OH)D3 and phosphorus increased in the two groups (Table 3). In multiple regression analysis, age, serum calcium, and baseline GFR were independent predictors of GFR variation. Parathyroid adenoma volume and urinary calcium were not independent predictors of GFR change. Multiple regression analyses are shown in Table 4.

**Table 1:** Clinical and biochemical pre- and postparathyroidectomy parameters in PHPT patients.

	Preparathyroidectomy(n = 125)	Postparathyroidectomy(n = 125)	P
BMI (kg/m2)	30.87 ± 5.22	31.06 ± 5.05	0.281
GFR (mL/min/1.73 m2)	91.41 ± 18.76	94.25 ± 17.46	0.08
Creatinine (mg/dL)	0.78 ± 0.21	0.75 ± 0.21	0.08
25(OH)D3 (ng/mL)	16.20 ± 17.97	32.47 ± 17.43	<0.001
Calcium (mg/dL)	11.13 ± 0.75	9.44 ± 0.77	<0.001
Phosphorous (mg/dL)	2.63 ± 0.44	3.26 ± 0.49	<0.001
PTH (pg/mL)	216.50 ± 148.77	63.49 ± 27.16	<0.001
ALP	115.38 ± 50.57	85.21 ± 28.29	<0.001
UA	5.35 ± 1.40	5.03 ± 1.33	0.01
Mg	2.14 ± 0.21	2.07 ± 0.20	0.01
Glucose (mg/dL)	95.88 ± 20.58	96.42 ± 33.40	0.56

Data are represented as mean ± SD.

**Table 2:** Clinical preparathyroidectomy parameters in PHPT patients according to GFR.

	GFR < 60 mL/min/1.73 m2(n = 16)	GFR ≥ 60 mL/min/1.73 m2(n = 109)	P
Age	66.12 ± 7.05	52.13 ± 9.93	<0.0001
Women, n (%)	12 (75)	94 (86)	0.27
Diabetics, n (%)	3 (23)	16 (14.6)	0.452
Hypertensives, n (%)	12 (75)	36 (33)	0.0014
Thiazide users, n (%)	2 (15)	22 (20)	0.672
Renal stones, n (%)	6 (40)	36 (33)	0.596
Renal cysts, n (%)	8 (53)	23 (22)	0.014
BMI (kg/m2)	32.67 ± 4.18	30.77 ± 5.33	0.11
Systolic BP (mmHg)	143.00 ± 16.76	137.68 ± 15.82	0.28
Diastolic BP (mmHg)	81.28 ± 7.68	84.52 ± 8.32	0.11
Creatinine (mg/dL)	1.17 ± 0.19	0.72 ± 0.14	<0.001
25(OH)D3 (ng/mL)	15.93 ± 12.55	16.23 ± 18.68	0.68
Calcium (mg/dL)	11.10 ± 0.60	11.13 ± 0.76	0.83
Phosphorous (mg/dL)	2.78 ± 0.45	2.60 ± 0.43	0.14
PTH (pg/mL)	154.38 ± 80.41	225.62 ± 154.46	0.01
ALP (U/L)	93.69 ± 31.70	118.56 ± 52.12	0.04
Uric acid (mg/dL)	7.30 ± 1.26	5.03 ± 1.14	<0.001
Mg (mg/dL)	2.03 ± 0.21	2.15 ± 0.21	0.08
Glucose (mg/dL)	96.80 ± 18.78	95.75 ± 20.90	0.96

Data are represented as mean ± SD.

**Table 3:** Clinical pre- and postparathyroidectomy parameters of PHPT patients according to GFR.

	GFR < 60 mL/min/1.73 m2 (n = 16)			GFR ≥ 60 mL/min/1.73 m2 (n = 109)		
	Pre-PTX	Post-PTX	P	Pre-PTX	Post-PTX	P
BMI (kg/m2)	32.67 ± 4.18	32.70 ± 4.40	0.05	30.77 ± 5.33	30.90 ± 5.11	0.35
GFR (mL/min/1.73 m2)	53.02 ± 5.94	58.42 ± 14.60	0.16	97.04 ± 12.20	98.53 ± 12.04	0.12
Creatinine (mg/dL)	1.1 ± 0.19	1.16 ± 0.31	0.56	0.72 ± 0.14	0.70 ± 0.13	0.10
25(OH)D3 (ng/mL)	15.9 ± 12.55	33.73 ± 15.52	0.001	16.23 ± 18.68	32.33 ± 17.69	<0.001
Calcium (mg/dL)	11.10 ± 0.60	9.43 ± 0.33	<0.001	11.13 ± 0.76	9.44 ± 0.48	<0.001
Phosphorous (mg/dL)	2.78 ± 0.45	3.36 ± 0.53	<0.001	2.60 ± 0.43	3.25 ± 0.49	<0.001
PTH (pg/mL)	154.38 ± 80.41	58.28 ± 20.80	0.001	225.62 ± 154.46	64.11 ± 27.83	<0.001
ALP	93.69 ± 31.59	76.27 ± 25.34	0.004	118.56 ± 52.12	86.11 ± 28.52	<0.001
UA	7.30 ± 1.26	6.89 ± 1.15	0.25	5.03 ± 1.14
4.78 ± 1.15	0.02	Mg	2.03 ± 0.21	2.11 ± 0.34	0.62	2.15 ± 0.21	2.07 ± 0.19	0.007
Glucose (mg/dL)	96.80 ± 18.78	87.63 ± 7.27	0.44	95.75 ± 20.90	97.31 ± 34.88	0.54

**Table 4:** Multiple linear regression model of independent predictors of variation in eGFR before and after parathyroidectomy in primary hyperparathyroidism (dependent variable = eGFR).

Independent variables	β	P-value
Parathyroid adenoma volume	0.041	0.66
Age (years)	0.436	<0.001
Baseline calcium (mg/dL)	–0.247	0.01
Baseline creatinine (mg/dL)	–0.001	0.99
Baseline GFR (mL/min/1.73 m2)	0.610	0.014

## 4. Discussion

The primary target for PHPT is the kidney. Impairment of renal function is an indication for surgery because of two concerns. The first concern is renal failure causing the progression in hyperparathyroidism, in which the mechanism can be explained by adding secondary hyperparathyroidism to already existing PHPT. The second concern is PHPT leading to deterioration in kidney function via nephrolithiasis/nephrocalcinosis or direct effects of serum calcium or PTH on renal function (9). Mild untreated PHPT increases mortality and morbidity, as seen in more severe PHPT (20). Current guidelines recommend parathyroidectomy in PHPT patients if their GFR is lower than 60 mL/min/1.73 m2 (8). The course of renal function in PHPT patients with concomitant renal disease remains controversial. Moreover, there is scarce evidence in the literature about mild PHPT negatively affecting renal function. A recent study demonstrated that the guideline’s recommendations regarding PHPT had not been followed, and renal impairment predicted against PTX in the study population (21). Tassone et al. demonstrated that renal function decreased after curative PTX in PHPT patients with GFR above 60 mL/min/1.73 m2; however, curative PTX ceased further renal impairment in PHPT patients with GFR lower than 60 mL/min/1.73 m2. Hence, they concluded that the decrease in renal function might not be affected by PTX (22). Our study shows that further renal impairment was prevented in both of our groups and presurgical CKD-EPI eGFR was significantly and independently associated with the variation in GFR after PTX. This result might suggest that parathyroidectomy can be recommended in PHPT patients regardless of renal function.

Renal function was deteriorated by prolonged hypercalcemia, and a reduced GFR is typical in advanced PHPT (3,4). Walker et al. investigated whether the levels of hypercalcemia and nephrolithiasis were associated with worse renal function in patients with PHPT or not, and they did not find that higher serum calcium or PTH or nephrolithiasis were related to worse renal function (23). In our present study, serum calcium was an independent predictor of GFR variation. Yamashita et al. (24) reported that the excised parathyroid weighed significantly more in the renal insufficiency group than in the normal renal function group. In our study, we could not find an association between parathyroid adenoma volume and renal function impairment. 

Prevalence of patients with hypertension and renal cysts was higher in patients with GFR lower than 60 mL/min/1.73 m2. These results might suggest that renal damage as a result of PHPT may lead to hypertension and renal cyst development.

Starup-Linde et al. reported that urinary calcium excretion was higher in PHPT patients with renal calcification than in PHPT patients without (25). Cassibba et al. (26) did not find a difference in total urinary calcium excretion among those who did or did not form kidney stones. Walker et al. (23) demonstrated a positive relationship between eGFR and urinary calcium excretion. Urinary calcium excretion was not an independent predictor of renal function impairment in our study. There are no data in the literature related to the change in urinary calcium excretion after parathyroidectomy and its possible role in affecting renal function deterioration in PHPT. However, in our present study, we did not investigate urinary calcium excretion after parathyroidectomy. 

Although it is not statistically significant, there is an increase in GFR in patients whose GFR is lower than 60 mL/min/1.73 m2. If we had more patients instead of only 16 patients in that group, it might have been possible to reach a statistically significant conclusion. On the other hand, more older patients and more patients with lower calcium levels could also increase the possibility of reaching statistically significance according to multiple linear regression analysis.

In conclusion, our findings indicate that older age, higher preoperative calcium, and GFR level were factors associated with GFR increase in PHPT patients after parathyroidectomy. Parathyroid adenoma volume and urinary calcium excretion are not associated with impairment of renal function. Further renal impairment was prevented by parathyroidectomy in PHPT patients. Moreover, parathyroidectomy might be recommended to all PHPT patients regardless of renal function.
